# Dietary flavonoids intake contributes to delay biological aging process: analysis from NHANES dataset

**DOI:** 10.1186/s12967-023-04321-1

**Published:** 2023-07-21

**Authors:** Wenmin Xing, Wenyan Gao, Zhenlei Zhao, Xiaogang Xu, Hongyan Bu, Huili Su, Genxiang Mao, Jun Chen

**Affiliations:** 1 Department of Geriatrics, Zhejiang Provincial Key Laboratory of Geriatrics, Zhejiang Hospital, No. 1229, Gudun Road, 310013 Hangzhou, China; 2 School of Pharmacy, Hangzhou Medical College, Hangzhou, China

**Keywords:** Flavonoids intake, Aging, Biological age, Heart biological age, NHANES

## Abstract

**Background:**

Diet may influence biological aging and the discrepancy (∆age) between a subject’s biological age (BA) and chronological age (CA). We aimed to investigate the correlation of dietary flavonoids with the ∆age of organs (heart, kidney, liver) and the whole body.

**Method:**

A total of 3193 United States adults were extracted from the National Health and Nutrition Examination Survey (NHANES) in 2007–2008 and 2017–2018. Dietary flavonoids intake was assessed using 24-h dietary recall method. Multiple linear regression analysis was performed to evaluate the association of dietary flavonoids intake with the ∆age of organs (heart, kidney, liver) and the whole body. BA was computed based on circulating biomarkers, and the resulting ∆age was tested as an outcome in linear regression analysis.

**Results:**

The ∆age of the whole body, heart, and liver was inversely associated with higher flavonoids intake (the whole body ∆age β = − 0.58, cardiovascular ∆age β = − 0.96, liver ∆age β = − 3.19) after adjustment for variables. However, higher flavonoids intake positively related to renal ∆age (β = 0.40) in participants with chronic kidney disease (CKD). Associations were influenced by population characteristics, such as age, health behavior, or chronic diseases. Anthocyanidins, isoflavones and flavones had the strongest inverse associations between the whole body ∆age and cardiovascular ∆age among all the flavonoids subclasses.

**Conclusion:**

Flavonoids intake positively contributes to delaying the biological aging process, especially in the heart, and liver organ, which may be beneficial for reducing the long-term risk of cardiovascular or liver disease.

**Supplementary Information:**

The online version contains supplementary material available at 10.1186/s12967-023-04321-1.

## Introduction

The average life expectancy has increased from 45 years in the early 1900s to approximately 80 years today due to advancements in medical technology [1]. As a result, the global prevalence of age-related diseases is expected to rise substantially, posing a growing burden on the healthcare system and society, particularly in China and other developing countries [2]. Thus, it is vital to explore how to extend the health span and delay aging [3].

Now, aging can be defined as the degenerative changes in the body’s tissue structure and physiological functions [4]. However, the progression of aging is not uniform due to individual differences. Therefore, aging is commonly indicated by biological age (BA), also known as physiological aging, which is influenced by genetic, environmental factors, lifestyle habits, and mental state [5, 6]. Recently, it has been a proposal to calculate biological age to assess the aging condition of the human body, as it provides a more accurate reflection of the body’s aging compared to chronological age (CA) [5]. Biological age is inferred from the developmental state of normal human physiology and anatomy, indicating the actual condition of the body’s tissue structure and physiological functions. BA is typically calculated using multiple parameters, such as blood biomarkers [7], DNA methylation [8], and structural neuroimaging measures [9].

As age increases, genetic factors, unhealthy lifestyles, such as smoking, alcohol consumption, chronic diseases, and cancer, accelerate aging and consequently increase biological age [10, 11]. Conversely, maintaining a healthy lifestyle, including moderate exercise and consuming vegetables and fruits, can decrease biological age [12]. For instance, the Mediterranean diet has been previously validated for its ability to slow down biological aging [13]. Individuals with high adherence to the Mediterranean diet had an average biological age almost one year younger than their chronological age, compared to those with low adherence [14]. Flavonoids are widely found in plants, fruits, and vegetables, and they can be classified into flavonoids, flavonols, isoflavones, flavones, flavanones, and anthocyanidins [13]. Moreover, flavonoids possess anti-inflammatory and antioxidant properties that benefit the body’s system [15, 16]. Studies have demonstrated that flavonoids can inhibit fat absorption and reduce lipogenesis, leading to normalized blood lipid levels [17]. Additionally, flavonoids have been shown to lower blood pressure by improving endothelial function [18, 19]. These characteristics may partially explain the observed benefits of consuming foods rich in flavonoids in delaying the aging process.

From this, we anticipate that flavonoids may decelerate the rate of biological age progression, although no research has definitively established a relationship between flavonoid consumption and biological age. We will confirm this association by parameterizing biological age, ∆age (∆age = BA − CA), and flavonoid intake based on analysis of National Health and Nutrition Examination Survey (NHANES) data and Food and Nutrient Database for Dietary Studies (FNDDS) data from 2007 to 2010 and 2017–2018.

## Methods

### Study population

The NHANES database is an annual cross-sectional survey conducted annually in the United States, comprising a health interview survey and a physical health survey of participants. All data collected from NHANES participants has been approved by the Ethics Review Board of the NCHS (available on the web at: https://www.cdc.gov/nchs/nhanes/). For this study, we analyzed flavonoids intake data from a total of 3,193 participants during the 2007–2010 and 2017–2018 NHANES cycles. Participants who were unable to complete dietary information interviews within 24 h were excluded, as well as pregnant women and cancer patients receiving medical or radiotherapy treatment [20]. Participant information regarding demographics, health-related lifestyle, and chronic conditions was collected, resulting in a final inclusion of 3913 individuals for the current study (Table 1).

**Table 1:** Characteristics of NHANES participants by tertile of total flavonoids intake

	Total population (n = 3913)	Total flavonoid intake tertiles			
		Q1	Q2	Q3	*P* value
Total flavonoid intake, mean(SE) (mg/d)	222.16 (11.04)	15.09 (0.35)	51.96 (0.85)	449.27 (15.15)	** < 0.01**
Socio-economic characteristics					
Total, n (%)	3913	1088 (26.35)	1174 (28.37)	1651 (45.28)	
Male, n (%)	1893 (48.38)	535 (27.02)	576 (29.31)	782 (43.67)	**0.41**
Female, n (%)	2020 (51.62)	553 (25.71)	598 (27.47)	869 (46.82)	
Age, years, mean (SD)	47.62 (0.44)	44.67 (0.63)	47.29 (0.76)	49.54 (0.65)	** < 0.0001**
Ethnicity					
White, n (%)	2002 (72.56)	547 (71.99)	547 (69.69)	908 (74.70)	**0.001**
Black, n (%)	655 (10.04)	202 (11.51)	188 (10.32)	265 (9.01)	
Mexican, n (%)	684 (7.62)	206 (8.65)	251 (10.03)	227 (5.52)	
Others, n (%)	572 (9.77)	133 (7.85)	188 (9.96)	251 (10.78)	
Healthy behavior factors					
Smoke status					
Current Smokers, n (%)	775 (18.72)	300 (26.23)	209 (17.67)	266 (15.01)	**0.001**
No current smokers, n (%)	3138 (81.28)	788 (73.77)	965 (82.33)	1385 (84.99)	
Drinking					
No drink user, n (%)	514 (10.70)	141 (10.60)	163 (12.67)	210 (9.51)	** < 0.0001**
Former drink user, n (%)	770 (15.95)	261 (19.81)	221 (14.62)	288 (14.55)	
Mild drink user, n (%)	1297 (37.51)	291 (29.35)	373 (34.81)	633 (43.96)	
Moderate drink user, n (%)	564 (15.96)	146 (16.53)	165 (15.06)	253 (16.20)	
Heavy drink user, n (%)	768 (19.87)	249 (23.71)	252 (22.84)	267 (15.78)	
Physical activity level					
Never, n (%)	1067 (21.45)	340 (23.83)	327 (21.96)	400 (19.76)	**0.33**
Low, n (%)	1039 (27.86)	252 (24.80)	304 (26.94)	483 (30.22)	
Intermediate, n (%)	942 (27.60)	258 (26.74)	281 (27.97)	403 (27.88)	
High, n (%)	865 (23.08)	238 (24.63)	262 (23.14)	365 (19.76)	
Dietary intake					
Kcal/day, kcal, mean (SD)	2103.68 (17.30)	1897.28 (34.24)	2168.36 (34.23)	2183.26 (24.75)	** < 0.0001**
Carbohydrates/day, g/100 kcal, mean (SD)	255.05 (1.91)	223.51 (3.91)	261.54 (3.95)	269.34 (2.91)	** < 0.0001**
Protein/day, g/100 kcal, mean (SD)	83.33 (0.88)	76.76 (1.83)	84.56 (1.40)	86.39 (1.28)	** < 0.001**
Fiber/day, g/100 kcal, mean (SD)	16.98 (0.31)	12.63 (0.24)	17.90 (0.29)	18.94 (0.47)	** < 0.0001**
Total fat, mean(SD)	79.99 (0.75)	74.76 (1.50)	81.03 (1.39)	82.37 (1.15)	** < 0.001**
Chronic disease factors					
BMI, kg/m^2^, n (%)					
BMI < 25	1117 (31.79)	296 (28.87)	333 (33.17)	488 (32.63)	**0.21**
BMI ≥ 25	2796 (68.21)	792 (71.13)	841 (66.83)	1163 (67.37)	
Hypertension, n (%)					
No Hypertension	2248 (63.57)	616 (63.20)	673 (63.87)	959 (63.60)	**0.96**
Hypertension	1665 (36.43)	472 (36.80)	501 (36.13)	692 (36.40)	
Diabetes, n (%)					
No diabetes	2356 (66.48)	629 (65.87)	714 (67.84)	1013 (65.97)	**0.58**
Diabetes	1557 (33.52)	459 (34.13)	460 (32.16)	638 (34.03)	
Chronic kidney disease					
No CKD	3232 (87.35)	883 (88.34)	965 (86.10)	1384 (87.55)	**0.42**
CKD	681 (12.65)	205 (11.66)	209 (13.90)	267 (12.45)	

NHANES, National Health and Nutrition Examination Survey; values are means (SD) for continuous variables and n (%) for categorical variables; SE: standard error; SD: standard deviation; BMI, body mass index; CKD, chronic kidney disease; MET, metabolic equivalent. Total flavonoids = sum of 29 individual flavonoids in six sub-classes: anthocyanidins, flavan-3-ols, flavanones, flavones, flavonols, and isoflavones. The total flavonoids were divided into Q1, Q2 and Q3 by tertile. Data were analyzed using χ^2^ tests for the categorical and ANOVA analysis for continuous variables

### Flavonoids intake

The intake information of flavonoids was gathered using the U.S. Department of Agriculture (USDA) Automated Multiple-Pass Method [21]. All collected foods were coded using the USDA FNDDS database, and then linked to specific flavonoid values using the Database of Flavonoid Values for USDA Survey Food Codes 2007–2010 and 2017–2018 (Flavonoid Database) [22]. The Flavonoid Database consists of six subclasses of flavonoids, namely anthocyanidins, flavan-3-ols, flavanones, flavones, flavonols, and isoflavones, encompassing a total of 29 different types of flavonoids. For this study, we defined total flavonoids intake as the sum of the mean values from these six subclasses [20].

### Calculation of biological age

To calculate biological age (BA), the NhanesR package developed by Jing Zhang in R Studio was utilized, as used by Jianmin Zhu et al. [23]. This study computed various BAs, including the whole body, cardiovascular, renal, and liver biological age [7, 24]. Cardiovascular biological age was calculated using markers like average diastolic and systolic blood pressure, fasting glucose, fasting total cholesterol, high-density cholesterol, low-density cholesterol, and fasting triglycerides. Renal biological age was derived from markers including uric acid, albumin, and creatinine. Liver biological age was computed using markers such as aspartate transaminase, alanine aminotransferase, albumin, and gamma-glutamyl transferase. The whole body biological age was calculated using an average of diastolic and systolic blood pressure, total cholesterol, alkaline phosphatase, creatinine, uric acid, total white blood cells, lymphocytes, hemoglobin, HbA1c, mean cell volume, and C-reactive protein. Finally, the ∆age for each BA (∆age = BA − chronological age) was calculated based on the individual’s BA and chronological age.

### Variables

The present study utilized various general variables during statistical analysis, including age, gender, race, smoking, alcohol consumption, BMI, and several chronic diseases. Participants were categorized into three groups: < 30 years, 30–59 years, and ≥ 60 years. Race was classified as White, Black, Mexican, and others. Overweight individuals were identified as those with a BMI ≥ 25 kg/m^2^ [25]. Alcohol users were categorized into the following groups based on a previous study [26]: ‘No drink user,‘ ‘Former drink user,‘ ‘mild drink user,‘ ‘Moderate drink user,‘ and ‘Heavy drink user’. Smoking status was defined as either former or current smoking. Physical activity was measured in weekly metabolic equivalent (MET) minutes. MET was divided into three sections (Q1: low, Q2: intermediate, and Q3: high), and participants were grouped as never, low, intermediate, and high levels of physical activity. Chronic diseases, such as hypertension, diabetes, and chronic kidney disease were also defined.

### Statistical analysis

The NHANES recommended weights were utilized to account for the planned oversampling of specific groups. Continuous variables were expressed as means ± standard deviations, while categorical variables were expressed as counts (percentages). Then, χ^2^ tests and one-way analysis of variance (ANOVA) were conducted to assess the associations between independent variables, each biological age (BA), and each ∆age. Furthermore, multiple linear regressions were performed to explore the associations between flavonoids intake and each ∆age while adjusting for covariates. Additionally, stratified analyses were performed according to age, gender, BMI, behavior, and chronic diseases (hypertension, diabetes, or CKD). All statistical analyses were conducted using the R software (version 4.1.2), RStudio software, and NhanesR package were used for.

## Results

### Basic clinical characteristics of study participants

A total of 3,193 participants were included in this study. The clinical characteristics of the study population for each independent variable are provided in Table 1. The distribution of total flavonoids and subclass intake into tertiles is described in Additional file 7: Table S1. The average age of the participants was 47.62 ± 0.44 years. The average total flavonoids intake was 222.16 ± 11.4 mg/day. Among the study population, 26.35% of participants were categorized in the lowest tertile of total flavonoids intake (15.09 ± 0.35 mg/day), while 45.28% were classified in the highest tertile of total flavonoids intake (449.27 ± 15.15 mg/day). A total of 1893 (48.38%) participants were male, while 2020 (51.62%) were female. The majority of participants were White ethnicity (72.56%). Compared to participants in the lowest tertile of total flavonoids intake, those in the highest tertile had a lower prevalence of current smokers and heavy alcohol users. Additionally, they were less likely to have hypertension, diabetes, or CKD but more likely to be overweight (BMI ≥ 25). Moreover, a higher flavonoids intake was associated with increased antioxidant capacity of the diet, as well as highera levels of fiber, total fat, protein, and carbohydrate intake (Table 1).

### Difference between biological age and chronological age in the study population

Table 2 presents the mean and standard deviation values of BA and the ∆age for each categorical variable. The whole body BA, calculated based on total flavonoids intake, was 49.75 years, with a ∆age of − 0.58 years. The cardiovascular BA was 46.65 years, with a ∆age of − 0.96 years. The renal BA was 48.01 years, with a ∆age of 0.40 years. The liver BA was 44.43 years, with a ∆age of − 3.19 years. Although the levels of dietary flavonoids intake were positively associated with kidney biological age, stratified analysis revealed that the biological age of individuals without CKD was 48, with a ∆age of − 0.62 years, while those with CKD had a biological age of 70, with a ∆age of 7.41 years. This indicates that CKD factors may impact the kidney biological age of the overall population. Compared to participants in the lowest tertile, participants in the middle and highest tertiles had a higher whole body, cardiovascular, and renal BA, but lower ∆age for whole body, cardiovascular, and liver (middle tertile: systemic ∆age (− 0.51), cardiovascular ∆age (− 0.55), liver ∆age (− 4.15), P value < 0.01); highest tertile: whole ∆age (− 1.10), cardiovascular ∆age (-1.68), liver ∆age (− 4.69), P value < 0.01). Similar results were observerd in the subclass of flavonoids intake. Participants in the highest tertile with anthocyanidins intake had the lowest ∆age for whole body BA (− 1.45), cardiovascular BA (− 1.87), renal BA (− 1.10), and liver BA (− 6.31) compared to those in the lowest tertile (Additional file 7: Table S2). Isoflavones and flavones also significantly contributed to delaying the whole body biographical aging and cardiovascular aging. In addition, flavones showed greater more effectiveness in delaying liver biographical aging than anthocyanidins (Additional file 7: Table S2).

**Table 2:** The averages of biological age (BA) and its differences with chronological age (CA) by tertile of total flavonoids intake

Variables	BA		BA difference (BA − CA)		Heart BA		Heart BA difference (BA − CA)		Kidney BA		Kidney BA difference (BA − CA)		Liver BA		Liver BA difference (BA − CA)	
	Mean (SD)	*P*	Mean (SD)	*P*	Mean (SD)	P	Mean (SD)	P	Mean (SD)	P	Mean (SD)	P	Mean (SD)	P	Mean (SD)	P
Total flavonoid intake tertiles	47.03 (0.49)	**–**	− 0.58 (0.15)	**–**	46.65 (0.49)	**–**	− 0.96 (0.23)	**–**	48.01 (0.59)	**–**	0.40 (0.34)	**–**	44.43 (0.88)	**–**	− 3.19 (0.71)	**–**
Q1	44.89 (0.72)	** < 0.01**	0.22 (0.28)	** < 0.01**	44.49 (0.73)	**0.01**	− 0.17 (0.32)	** < 0.01**	45.83 (0.85)	**0.01**	1.17 (0.66)	**0.36**	45.11 (1.38)	**0.52**	0.44 (1.17)	** < 0.01**
Q2	46.79 (0.85)		− 0.51 (0.25)		46.74 (0.87)		− 0.55 (0.43)		47.45 (0.92)		0.15 (0.49)		43.14 (1.64)		− 4.15 (1.11)	
Q3	48.43 (0.66)		− 1.10 (0.16)		47.86 (0.69)		− 1.68 (0.29)		49.64 (0.77)		0.10 (0.47)		44.84 (1.01)		− 4.69 (0.79)	
Sex																
Male	47.36 (0.54)	**0.29**	0.91 (0.16)	** < 0.01**	46.47 (0.50)	**0.58**	0.02 (0.30)	** < 0.01**	53.91 (0.68)	** < 0.01**	7.46 (0.35)	** < 0.01**	38.82 (1.14)	** < 0.01**	− 7.63 (0.85)	** < 0.01**
Female	46.72 (0.60)		− 2.01 (0.18)		46.83 (0.66)		− 1.90 (0.27)		42.40 (0.65)		− 6.33 (0.33)		49.77 (0.97)		1.04 (0.87)	
Age (years)																
~ 30	25.53 (0.35)	** < 0.01**	0.82 (0.29)	** < 0.01**	25.23 (0.38)	** < 0.01**	0.53 (0.31)	** < 0.01**	27.69 (0.69)	** < 0.01**	2.98 (0.65)	** < 0.01**	18.94 (1.24)	** < 0.01**	− 5.77 (1.21)	** < 0.01**
30–59	43.86 (0.30)		− 1.02 (0.18)		43.56 (0.32)		− 1.33 (0.29)		44.64 (0.50)		− 0.25 (0.42)		42.72 (1.07)		− 2.16 (0.94)	
≥ 60	69.21 (0.37)		− 0.61 (0.23)		68.60 (0.59)		− 1.22 (0.44)		69.80 (0.55)		− 0.02 (0.52)		66.19 (1.00)		− 3.63 (0.99)	
Ethnicity																
White	48.57 (0.58)	** < 0.01**	− 0.56 (0.41)	** < 0.01**	47.95 (0.58)	** < 0.01**	− 1.53 (0.30)	** < 0.01**	49.79 (0.73)	** < 0.01**	0.31 (0.40)	** < 0.01**	44.17 (1.20)	** < 0.01**	− 5.31 (0.95)	** < 0.01**
Black	45.51 (0.87)		1.05 (0.40)		46.00 (1.06)		1.54 (0.58)		47.21 (1.14)		2.75 (0.75)		51.90 (1.74)		7.44 (1.58)	
Mexican	40.99 (0.80)		0.31 (0.31)		41.73 (0.93)		1.06 (0.48)		39.27 (0.94)		− 1.40 (0.58)		43.50 (1.17)		2.83 (1.00)	
Others	41.87 (1.05)		− 0.91 (0.20)		41.51 (1.19)		− 0.93 (0.48)		42.43 (1.25)		− 0.01 (0.97)		39.43 (2.06)		− 3.01 (1.73)	
Healthy behavior factors																
Smoke status																
Current smokers	42.61 (0.81	** < 0.01**	0.58 (0.22)	** < 0.01**	41.67 (0.88)	** < 0.01**	− 0.36 (0.37)	**0.08**	42.25 (0.89)	** < 0.01**	0.21 (0.71)	**0.78**	43.49 (1.89)	**0.6**	1.46 (1.39)	** < 0.01**
No smoke	48.05 (0.57)		− 0.85 (0.15)		47.80 (0.59)		− 1.10 (0.25)		49.34 (0.71)		0.44 (0.38)		44.65 (1.00)		− 4.26 (0.79)	
Drinking																
No drink user	51.29 (1.06)	** < 0.01**	− 0.20 (0.28)	** < 0.01**	51.19 (1.00)	** < 0.01**	− 0.30 (0.50)	** < 0.01**	49.42 (1.22)	** < 0.01**	− 2.07 (0.67)	** < 0.01**	55.17 (2.12)	** < 0.01**	3.68 (1.62)	** < 0.01**
Former	54.54 (0.99)		− 0.20 (0.28)		54.29 (1.04)		− 0.45 (0.40)		53.88 (1.22)		− 0.86 (0.69)		55.09 (1.94)		0.35 (1.48)	
Mild	49.03 (0.60)		− 1.35 (0.19)		48.44 (0.64)		− 1.94 (0.37)		51.41 (0.76)		1.03 (0.41)		42.78 (1.24)		− 7.60 (0.93)	
Moderate	42.79 (0.89)		− 1.28 (0.34)		42.44 (0.98)		− 1.62 (0.51)		42.76 (1.02)		− 1.30 (0.64)		41.13 (1.44)		− 2.93 (1.34)	
Heavy	38.36 (0.64)		0.91 (0.28)		38.09 (0.72)		0.64 (0.45)		40.35 (0.80)		2.90 (0.66)		35.84 (1.63)		− 1.61 (1.51)	
Physical activity level																
Never	54.30 (0.70)	** < 0.01**	0.10 (0.25)	** < 0.01**	53.57 (0.73)	** < 0.01**	− 0.63 (0.35)	**0.01**	54.25 (0.85)	** < 0.01**	0.05 (0.55)	** < 0.01**	58.87 (1.66)	** < 0.01**	4.67 (1.43)	** < 0.01**
Low	48.43 (0.72)		− 1.21 (0.26)		48.13 (0.81)		− 1.50 (0.46)		48.75 (0.87)		− 0.88 (0.56)		46.54 (1.16)		− 3.09 (0.97)	
Intermediate	43.98 (0.75)		− 1.19 (0.24)		43.73 (0.78)		− 1.44 (0.36)		45.42 (0.95)		0.25 (0.62)		36.52 (1.07)		− 8.65 (0.84)	
High	42.24 (0.75)		0.26 (0.27)		41.94 (0.73)		− 0.04 (0.41)		44.43 (0.97)		2.44 (0.48)		37.92 (1.23)		− 4.07 (0.99)	
Chronic disease factors																
BMI, kg/m^2^																
BMI < 25	42.63 (0.84)	** < 0.01**	− 2.12 (0.25)	** < 0.01**	41.63 (0.85)	** < 0.01**	− 3.12 (0.32)	** < 0.01**	41.21 (0.84)	** < 0.01**	− 3.54 (0.53)	** < 0.01**	33.53 (1.35)	** < 0.01**	− 11.22 (0.95)	** < 0.01**
BMI ≥ 25	49.08 (0.36)		0.13 (0.15)		49.00 (0.40)		0.04 (0.25)		51.19 (0.56)		2.23 (0.38)		49.51 (0.85)		0.56 (0.84)	
Hypertension																
No Hypertension	40.00 (0.49)	** < 0.01**	− 1.81 (0.17)	** < 0.01**	38.93 (0.49)	** < 0.01**	− 2.88 (0.27)	** < 0.01**	40.79 (0.64)	** < 0.01**	− 1.02 (0.36)	** < 0.01**	36.47 (0.91)	** < 0.01**	− 5.33 (0.77)	** < 0.01**
Hypertension	59.31 (0.44)		1.55 (0.20)		60.14 (0.45)		2.38 (0.30)		60.62 (0.66)		2.86 (0.54)		58.32 (1.09)		0.56 (1.03)	
Diabetes																
No diabetes	41.52 (0.54)	** < 0.01**	− 1.59 (0.18)	** < 0.01**	40.70 (0.53)	** < 0.01**	− 2.41 (0.25)	** < 0.01**	42.78 (0.68)	** < 0.01**	− 0.33 (0.36)	** < 0.01**	38.00 (1.04)	** < 0.01**	− 5.10 (0.83)	** < 0.01**
Diabetes	57.97 (0.55)		1.40 (0.22)		58.47 (0.57)		1.90 (0.34)		58.40 (0.73)		1.83 (0.55)		57.18 (1.09)		0.61 (1.08)	
Chronic kidney disease																
No CKD	44.33 (0.51)	** < 0.01**	− 1.10 (0.16)	** < 0.01**	44.06 (0.50)	** < 0.01**	− 1.36 (0.24)	** < 0.01**	44.81 (0.59)	** < 0.01**	− 0.62 (0.33)	** < 0.01**	41.36 (0.94)	** < 0.01**	− 4.07 (0.71)	** < 0.01**
CKD	65.70 (0.95)		2.96 (0.37)		64.54 (1.05)		1.80 (0.65)		70.15 (1.35)		7.41 (1.04)		65.64 (1.78)		2.91 (1.66)	

Values are means (SD) for continuous variables. Data were analyzed using ANOVA analysis for continuous variables

*SD* standard deviation, *BMI* body mass index, *CKD* chronic kidney disease.

Females were more likely to have a lower whole body, cardiovascular, and renal ∆age but a higher liver ∆age (P value < 0.01) compared to males. Health behavior indicators, including physical activity, smoking, and alcohol consumption, also influence BA and ∆age. For instance, smoking, heavy alcohol consumption, and high levels of physical activity were associated with higher ∆age for the whole body, cardiovascular, and renal BA. However, heavy alcohol consumption and a high level of physical activity appeared to have a lower ∆age for liver BA (P value < 0.01). Higher BMI was associated with higher BA and ∆age for the whole body BA, cardiovascular BA, renal BA, and liver BA (P value < 0.01). Participants with chronic diseases, such as hypertension, diabetes, showed a similar trend in BA and ∆age.

### Association between flavonoids intake and biological age

Linear regression analysis was conducted to examine the association between flavonoid intake and ∆age and the results are summarized in Table 3. Participants in the middle tertile and highest tertile of total flavonoids intake showed an inverse correlation with ∆age (middle tertile β = − 0.73, P value < 0.01, highest tertile β = − 1.33, P value < 0.01, ref = the lowest tertile). Similar results were observed for cardiovascular BA, renal BA, and liver BA. Moreover, subclass flavonoids intake also exhibited an inverse trend with ∆age, particularly in the liver (Additional file 7: Table S3). Females and individuals in the 30–59 age group had lower ∆ages compared to other groups. Conversely, participants engaging in unhealthy behavior, such as smoking or alcohol consumption, demonstrated a positive association with higher ∆age. Similarly, individuals with chronic diseases, such as hypertension, diabetes, obesity (BMI ≥ 25), or CKD, exhibited a positive correlated with higher ∆age.

**Table 3:** The results of multiple linear regression analysis for the association between total flavonoids intake and differences in biological age (BA) with chronological age (CA)

Variables	Whole body BA differences		Heart BA differences		Kidney BA differences		Liver BA differences	
	β(95%CI)	P	β(95%CI)	P	β(95%CI)	P	β(95%CI)	P
Total flavonoid intake								
Q1	Ref.	**–**	Ref.	**–**	Ref.	**–**	Ref.	**–**
Q2	− 0.73 (− 1.30, − 0.16)	**0.01**	− 0.38 (− 1.36, 0.60)	**0.43**	− 1.02 (− 2.62, 0.59)	**0.21**	− 4.59 (− 7.38, − 1.81)	** < 0.01**
Q3	− 1.33 (− 1.95, − 0.71)	** < 0.01**	− 1.51 (− 2.32, − 0.70)	** < 0.01**	− 1.07 (− 2.66, 0.52)	**0.18**	− 5.14 (− 7.77, − 2.50)	** < 0.01**
Sex								
Male	Ref.	**–**	Ref.	**–**	Ref.	**–**	Ref.	**–**
Female	− 2.92 (− 3.29, − 2.54)	** < 0.01**	− 1.92 (− 2.63, − 1.21)	** < 0.01**	− 13.79 (− 14.67, − 12.91)	** < 0.01**	8.66 (6.64, 10.68)	** < 0.01**
Age (years)								
~ 30	1.43 (0.80, 2.06)	** < 0.01**	1.74 (0.73, 2.75)	** < 0.01**	3 (1.35, 4.66)	** < 0.01**	− 2.14 (− 4.94, 0.67)	**0.13**
30–59	− 0.41 (− 0.98, 0.15)	**0.15**	− 0.11 (− 1.18, 0.96)	**0.84**	− 0.23 (− 1.30, 0.85)	**0.67**	1.47 (− 0.77, 3.71)	**0.19**
≥ 60	Ref.	**–**	Ref.	**–**	Ref.	**–**	Ref.	**–**
Healthy behavior factors								
Smoke status								
Current smokers	1.43 (0.99, 1.88)	** < 0.01**	0.74 (− 0.08, 1.57)	**0.08**	− 0.22 (− 1.82, 1.37)	**0.02**	5.71 (2.58, 8.84)	** < 0.01**
No smoke	Ref.	**–**	Ref.	**–**	Ref.	**–**	Ref.	**–**
Drinking								
No drink user	Ref.	**–**	Ref.	**–**	Ref.	**–**	Ref.	**–**
Former	0.01 (− 0.79, 0.80)	**0.99**	− 0.15 (− 1.62, 1.32)	**0.83**	1.21 (− 0.59, 3.01)	**0.18**	− 3.32 (− 7.83, 1.18)	**0.14**
Mild	− 1.15 (− 1.78, − 0.52)	** < 0.01**	− 1.64 (− 2.82, − 0.46)	**0.01**	3.1 (1.61, 4.60)	** < 0.01**	− 11.27 (− 14.59, − 7.95)	** < 0.01**
Moderate	− 1.07 (− 1.80, − 0.34)	**0.01**	− 1.32 (− 2.66, 0.01)	**0.05**	0.77 (− 0.84, 2.38)	**0.34**	− 6.61 (− 10.37, − 2.85)	** < 0.01**
Heavy	1.12 (0.27, 1.97)	**0.01**	0.94 (− 0.34, 2.22)	**0.14**	4.97 (2.96, 6.98)	** < 0.01**	− 5.28 (− 9.47, − 1.10)	**0.02**
Physical activity level								
Never	Ref.	**–**	Ref.	**–**	Ref.	**–**	Ref.	**–**
Low	− 1.31 (− 2.00, − 0.61)	** < 0.01**	− 0.87 (− 2.05, 0.31)	**0.14**	− 0.93 (− 2.40, 0.55)	**0.21**	− 7.76 (− 11.12, − 4.39)	** < 0.01**
Intermediate	− 1.29 (− 1.99, − 0.58)	** < 0.01**	− 0.81 (− 1.94, 0.32)	**0.15**	0.2 (− 1.40, 1.80)	**0.80**	− 13.32 (− 16.41, − 10.22)	** < 0.01**
High	0.16 (− 0.69, 1.01)	**0.70**	0.59 (− 0.70, 1.87)	**0.36**	2.39 (0.94, 3.84)	**0.002**	− 8.74 (− 11.60, − 5.87)	** < 0.01**
Chronic disease factors								
BMI, kg/m^2^								
BMI < 25	Ref.	**–**	Ref.	**–**	Ref.	**–**	Ref.	**–**
BMI ≥ 25	2.25 (1.70, 2.80)	** < 0.01**	3.17 (2.43, 3.91)	** < 0.01**	5.78 (4.68, 6.87)	** < 0.01**	11.78 (9.45, 14.11)	** < 0.01**
Hypertension								
No Hypertension	Ref.	**–**	Ref.	**–**	Ref.	**–**	Ref.	**–**
Hypertension	3.35 (2.87,3.83)	** < 0.01**	5.26 (4.51, 6.01)	** < 0.01**	3.88 (2.74, 5.01)	** < 0.01**	5.89 (3.63, 8.16)	** < 0.01**
Diabetes								
No diabetes	Ref.	**–**	Ref.	**–**	Ref.	**–**	Ref.	**–**
Diabetes	2.99 (2.42, 3.56)	** < 0.01**	4.3 (3.56, 5.05)	** < 0.01**	2.16 (1.00, 3.32)	** < 0.01**	5.71 (3.14, 8.28)	** < 0.01**
Chronic kidney disease								
No CKD	Ref.	**–**	Ref.	**–**	Ref.	**–**	Ref.	**–**
CKD	4.06 (3.19, 4.92)	** < 0.01**	3.16 (1.73,4.59)	** < 0.01**	8.03 (5.85, 10.21)	** < 0.01**	6.98 (3.75, 10.20)	** < 0.01**

Linear regression analysis was used to determine the standard β coefficient

*BMI* body mass index, *CKD* chronic kidney disease, *CI* confidence interval

**Fig. 1:**
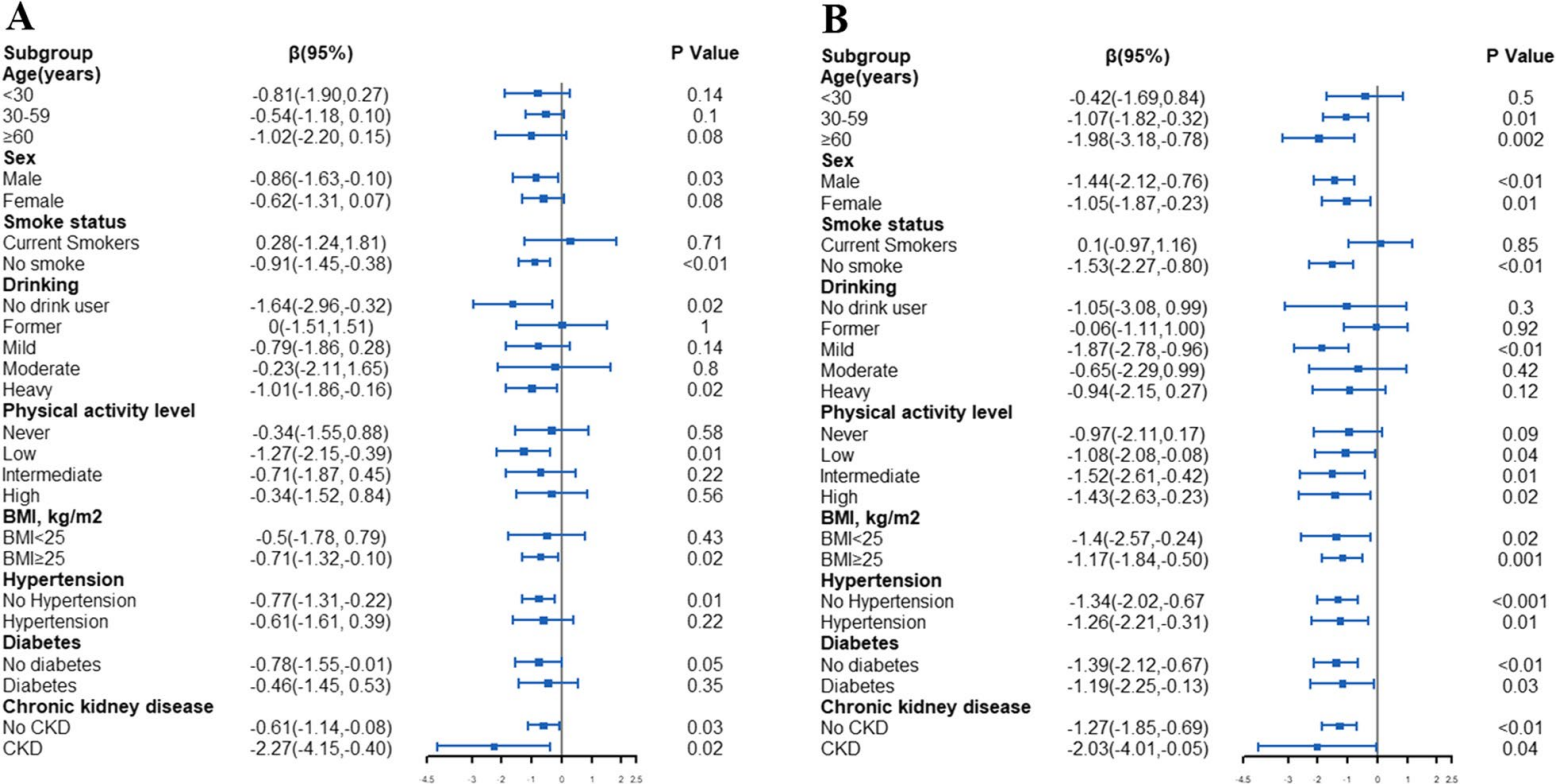
The results of stratified analysis for the association between flavonoid intake and the whole body ∆age according to different variables. **A** The association between the middle tertile of total flavonoids intake and the whole body ∆age. **B** The association between the highest tertile of total flavonoids intake and the whole body ∆age

Then, we performed stratified analysis using linear regression to examine the relationship between total flavonoids intake and ∆age across different variables, including age, gender, smoke status, alcohol consumption, physical activity level, and chronic disease diagnosis. The results are presented in Fig. 1 and Additional files 1, 2, 3, 4, 5, 6: Figs. S1–S6. We observed a higher prevalence of lower ∆age for the whole body BA among participants in the highest tertile of total flavonoids intake in the following groups: those aged > 30 years, no smoking group, mild alcohol consuming group, and exercise group, compared to others. Additionally, this prevalence was found in the 30–59 age group, males who were non-smokers, and had low level of exercise, and did’t have hypertension and diabetes group, while being in the middle tertile of total flavonoids intake (Fig. 1). Regarding heart BA, no significant difference was observed in ∆age between participants in the middle tertile of total flavonoids intake and various variables, such as age, gender, and health behavior (Additional file 1: Fig. S1). However, for participants in the highest tertile of total flavonoids intake, there was an inverse association between total flavonoids intake and ∆age in the following groups: those aged > 30 years, non-smokers, former alcohol consumers, individuals with intermediate exercise group, and those withouto diabetes or CKD, compared to other groups (Additional file 2: Fig. S2). Similar correlations were found for liver ∆age (Additional files 3, 4: Figs. S3–S4). However, no significant associations were confirmed between renal ∆age and total flavonoids among participants in the middle tertile or highest tertile of participants (Additional files 5, 6: Figs. S5–S6).

## Discussion

The escalating epidemic of population aging imposes a significant and growing burden on public health. Consequently, there is a critically important unmet need to address aging through behavioral and dietary modifications. In this cross-sectional study involving 3193 participants, a higher intake of total flavonoids was found to be associated with a lower biological age of the cardiovascular, hepatic, and whole body among White participants who did not smoke, consume alcohol, and were free from chronic diseases.

Our results were in alignment with the previous research, which has demonstrated an inverse association between biological aging and high adherence to the Mediterranean diet, which is abundant in polyphenol-rich foods [14]. Similarly, a cross-sectional analysis conducted as part of the Moli-sani Study (2005–2010) revealed an inverse association between a polyphenol antioxidant content (PAC)-score and biological age, determined through a deep neural network based on 36 circulating biomarkers [13]. Earlier studies have illustrated the beneficial relationship between polyphenol-rich diets and various markers of aging, including telomere length [27], cognitive decline [28], and DNA methylation [29]. These effects are thought to be attributed to the the antioxidant and anti-inflammatory properties of plant-based diets.

However, individuals who are aging at a faster rate may have difficulty identifying which specific organ(s) in their body are experiencing abnormalities and how to address them. Therefore, we conducted an investigation into the biological age of organs, such as the cardiovascular, renal, and liver biological age. Moreover, this study revealed variations in the rate of aging among different organs and the whole body. Consequently, flavonoids intake showed distinct associations with organic and whole body biological age. Specifically, a higher intake of flavonoids was inversely associated with the whole body ∆age, cardiovascular ∆age, and liver ∆age, but positively correlated with renal ∆age.

Moreover, gender played a significant role in biological aging. Specifically, females with higher flavonoids intake were inversely associated with whole body ∆age, cardiovascular ∆age, and renal ∆age, but positively related with liver ∆age. Interestingly, there was no significant difference in flavonoids intake between males and females. Consequently, we performed a stratified analysis based on sex, focusing on individuals in the middle and highest tertile of total flavonoid intake. The results indicated that higher flavonoids intake was more effective in delaying liver aging among females (middle tertile: liver ∆age, males β = − 1.49, females β = − 7.66; 75th : liver ∆age, males β = − 4.53, females β = − 6.31).

The researchers considered that the antioxidant properties of flavonoids are the main reasons for their potential anti-aging effects [30]. For instance, a previous analysis of NHANES data demonstrated significant negative associations between fatty liver index (FLI), serum aspartate aminotransferase (AST), alanine aminotransferase (ALT), and flavonoids intake in patients with non-alcoholic fatty liver disease (NAFLD) [31]. Moreover, there was stepwise decrease in the risk of NAFLD with increasing flavonoid intake (odds ratio: 0.81). C-reactive protein (CRP) strongly regulated the impact of flavonoids intake on FLI, which reduced the benefits of flavonoids intake. Mechanically, flavonoids inhibit oxidative stress and decrease oxidative damage through their hydroxyl groups’ free radical scavenging activity [32]. Simultaneously, the total antioxidant capacity of flavonoids intake potentially contributed to decreased serum triglyceride (TG). Moreover, flavonoid compounds improved heat, oxidative, and pathogenic stress resistances to delay the aging of *C. elegans.* This was achieved through the activation of stress responses, including HSF-1-mediated heat shock response, SKN-1-mediated xenobiotic and oxidation response, mitochondria unfolded response, endoplasmic unfolded protein response, and increased autophagy activity [33].

In the present study, anthocyanidins exhibited the strongest inverse associations between the whole body ∆age and cardiovascular ∆age among all the flavonoid subclasses. Isoflavones and flavones had the second most significant impact on delaying age-related changes in the whole body aging and cardiovascular aging. Additionally, flavones demonstrated greater effectiveness in delaying biological age in the liver compared to anthocyanidins. This is the first study to demonstrate dose–response relationships between different subclasses of flavonoids and biological age. The anti-aging effect of anthocyanidins can be comprehensively interpreted by numerous previous studies. For example, higher intakes of total anthocyanins were independently associated with a 32% lower rate of myocardial infarction, an 8–10% reduction in the risk of hypertension, and a 26% reduction in the risk of type 2 diabetes mellitus (T2DM) [34–36]. Similar to other flavonoids, anthocyanins contribute to overall health primarily through antioxidant and anti-inflammatory properties [37] to improve plasma lipid levels, modulate glucose metabolism and NO metabolism [38, 39], and maintain endothelial function [40].

### Strengths and limitations

This study possesses several strengths compared to previous findings. Firstly, it utilized biological age (BA) as the outcome variable to assess the impact of flavonoids intake on whole body aging and organic aging. Secondly, higher flavonoids intake was found to be effectively slow down the rate of whole body aging, cardiovascular aging, and liver aging, providing implications for the prevention of cardiovascular and liver diseases. Finally, this study was based on a large population-based survey (NHANES) dataset, which employed a strictly random sampling process, ensuring that our results are representative of the entire population.

However, our study does have some limitations that should also be acknowledged. Firstly, it employed a cross-sectional study design, which precludes establishing causal or temporal associations between flavonoids intake and whole body aging or organic aging. Secondly, there were inaccuracies in measuring dietary intake based on the 24-h dietary recall interviews, although we excluded participants who could not be interviewed. Thirdly, we did not account for other foods or nutrient supplements with antioxidating features, which may have influence the study results. Fourthly, the potential impact of medications on the results was not considered, leading to potential bias in our conclusions. Finally, BA was determined by several blood biomarkers, which may not be accurately represent other assessments of biological aging, such as brain age, telomere length, and DNA methylation [41–44]. However, these markers are costly and not feasible for most individuals.

## Conclusions

The results from this NHANES cohort demonstrated that higher flavonoids intake is associated with a deceleration of whole body biological aging, as well as cardiovascular and liver biological aging. This is the first population-based study to investigate the link between flavonoids intake and whole body and organic biological aging using a blood-based biomarker for biological age. Notably, anthocyanins, flavones, and isoflavones showed the strongest protective associations with whole body biological aging, cardiovascular biological aging, and liver biological aging. These findings suggest potential future interventions in aging, cardiovascular disease, or liver disease. It is possible that diets rich in flavonoids may be beneficial in delaying aging and promoting overall health.

## Supplementary Information

**Additionalfile 1: Figure S1. **Theresults of stratified analysis for the association between the middle tertileof flavonoids intake and the heart ∆age according todifferent variables.

**Additionalfile 2: Figure S2. **The results of stratified analysis for the associationbetween the highest tertile offlavonoids intake and the heart ∆age according to different variables.

**Additionalfile 3: Figure S3. **The results of stratified analysis for the associationbetween the middle tertile of flavonoidsintake and the kidney ∆ageaccording to different variables.

**Additionalfile 4: Figure S4. **The results of stratified analysis for the associationbetween the highest tertile offlavonoids intake and the kidney ∆age according to different variables.

**Additionalfile 5: Figure S5. **The results of stratified analysis for the associationbetween the middle tertile of flavonoidsintake and the liver ∆age according to different variables.

**Additionalfile 6: Figure S6.** The results of stratified analysis for theassociation between the highest tertile of flavonoids intake and the liver ∆age according to differentvariables.

**Additional file 7: Table S1**. Characteristics of NHS Participants by Quintiles of Total Flavonoid Intake. **Table S2. **The averages of biological age (BA) and its differences with chronological age (CA) by quintiles of total flavonoid intake. **Table S3 **The results of multiple linear regression analysis for the association between flavonoid intake and differences in biological age (BA) with chronological age (CA).

## Data Availability

The dataset(s) supporting the conclusions of this article is available through email the corresponding author.
