# Supplementary figures and images for: Dietary flavonoids intake contributes to delay biological aging process: analysis from NHANES dataset
Source: J Transl Med. 2023 Jul 21;21:492. doi: 10.1186/s12967-023-04321-1 (PMC10362762; doi:10.1186/s12967-023-04321-1)

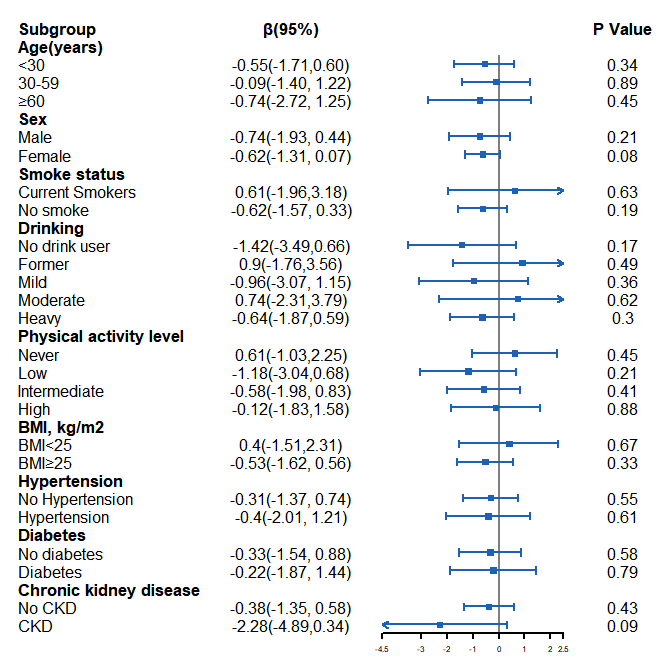

Supplement: Supplementary file 1 — Additionalfile 1: Figure S1. Theresults of stratified analysis for the association between the middle tertileof flavonoids intake and the heart ∆age according todifferent variables. [file 12967_2023_4321_MOESM1_ESM.tiff]

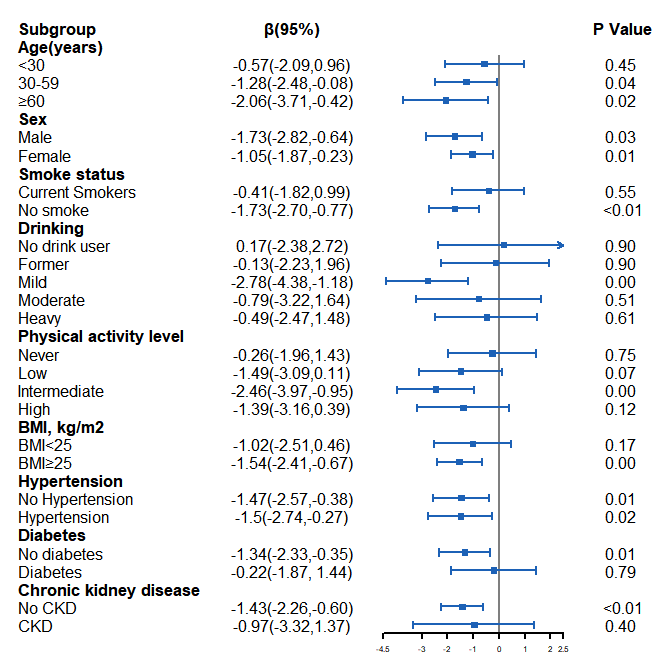

Supplement: Supplementary file 2 — Additionalfile 2: Figure S2. The results of stratified analysis for the associationbetween the highest tertile offlavonoids intake and the heart ∆age according to different variables. [file 12967_2023_4321_MOESM2_ESM.tiff]

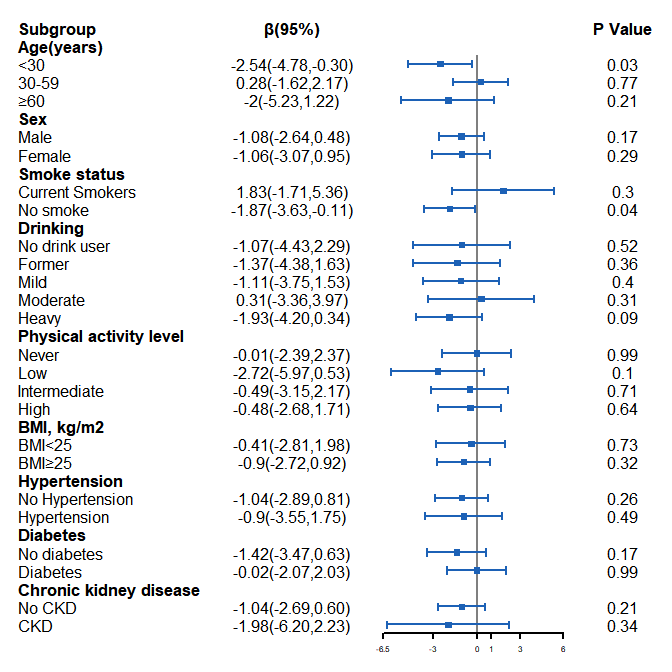

Supplement: Supplementary file 3 — Additionalfile 3: Figure S3. The results of stratified analysis for the associationbetween the middle tertile of flavonoidsintake and the kidney ∆ageaccording to different variables. [file 12967_2023_4321_MOESM3_ESM.tiff]

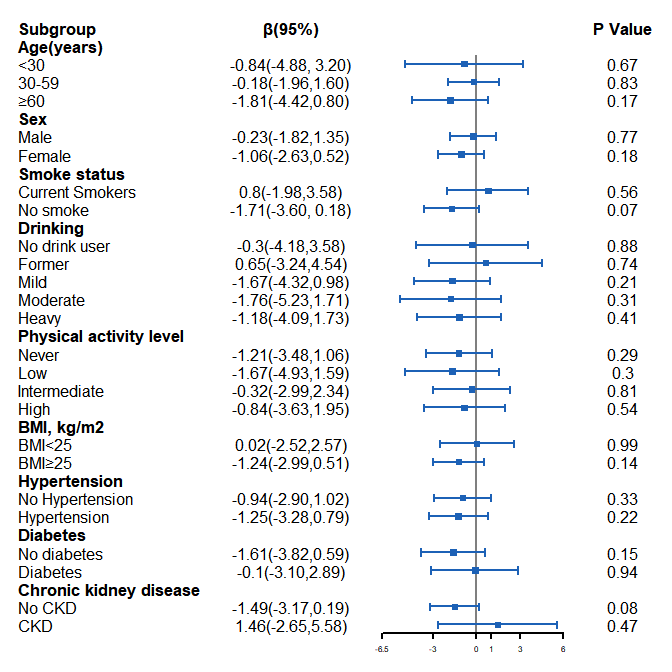

Supplement: Supplementary file 4 — Additionalfile 4: Figure S4. The results of stratified analysis for the associationbetween the highest tertile offlavonoids intake and the kidney ∆age according to different variables. [file 12967_2023_4321_MOESM4_ESM.tiff]

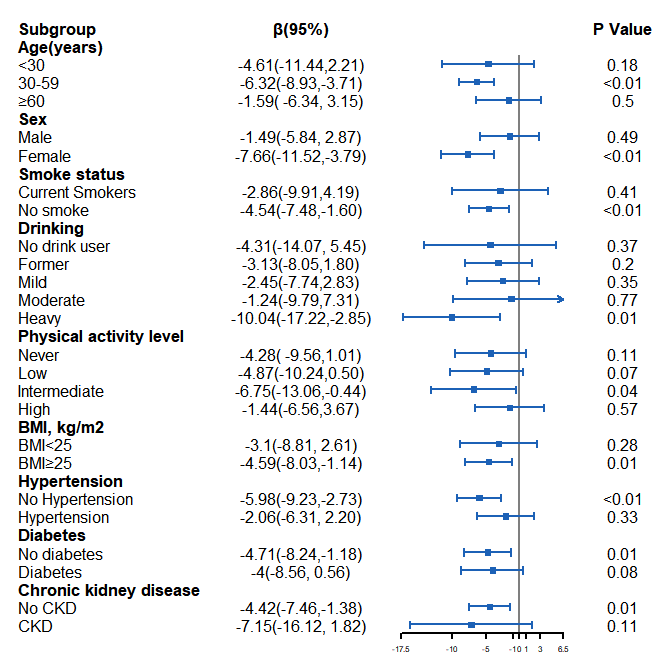

Supplement: Supplementary file 5 — Additionalfile 5: Figure S5. The results of stratified analysis for the associationbetween the middle tertile of flavonoidsintake and the liver ∆age according to different variables. [file 12967_2023_4321_MOESM5_ESM.tiff]

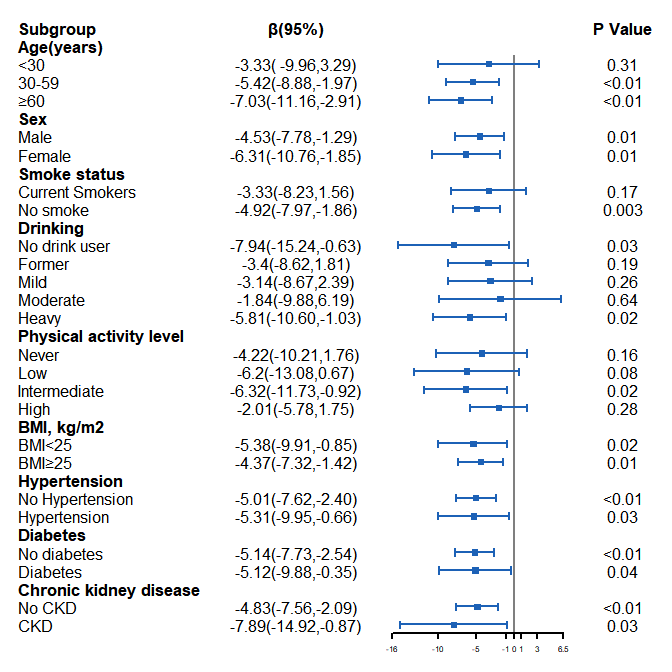

Supplement: Supplementary file 6 — Additionalfile 6: Figure S6. The results of stratified analysis for theassociation between the highest tertile of flavonoids intake and the liver ∆age according to differentvariables. [file 12967_2023_4321_MOESM6_ESM.tiff]
