# Supplementary material for: Dietary flavonoids intake contributes to delay biological aging process: analysis from NHANES dataset
Source: J Transl Med. 2023 Jul 21;21:492. doi: 10.1186/s12967-023-04321-1 (PMC10362762; doi:10.1186/s12967-023-04321-1)
Supplement: Supplementary file 7 — Additional file 7: Table S1. Characteristics of NHS Participants by Quintiles of Total Flavonoid Intake. Table S2. The averages of biological age (BA) and its differences with chronological age (CA) by quintiles of total flavonoid intake. Table S3 The results of multiple linear regression analysis for the association between flavonoid intake and differences in biological age (BA) with chronological age (CA). [file 12967_2023_4321_MOESM7_ESM.docx]

**Table S1. Characteristics of NHS Participants by Quintiles of Total Flavonoid Intake**

|  | **Total population** | **Total flavonoid intake quintiles** | | |  |
| --- | --- | --- | --- | --- | --- |
|  |  | **Q1** | **Q2** | **Q3** | **P value** |
| **Total_29_flavonoids** | **222.16(11.04)** | **3.78(0.06)** | **9.75(0.06)** | **29.24(0.66)** | **< 0.0001** |
| **Isoflavones** | **1.98(0.22)** | **0.00(0.00)** | **0.01(0.00)** | **5.41(0.61)** | **< 0.0001** |
| **Anthocyanidins** | **13.17(0.95)** | **0.09(0.00)** | **2.28(0.05)** | **31.59(1.72)** | **< 0.0001** |
| **Flavan_3_ols** | **174.68(10.32)** | **2.97(0.09)** | **14.42(0.19)** | **399.55(16.38)** | **< 0.0001** |
| **Flavanones** | **12.55(0.60)** | **0.02(0.00)** | **1.39(0.06)** | **38.30(1.04)** | **< 0.0001** |
| **Flavones** | **0.96(0.06)** | **0.08(0.00)** | **0.39(0.01)** | **1.72(0.12)** | **< 0.0001** |
| **Flavonols** | **18.82(0.56)** | **3.78(0.06)** | **9.75(0.06)** | **29.24(0.66)** | **< 0.0001** |
| **Daidzein, mean (SE), mg/d** | **0.77(0.09)** | **-** | **-** | **-** | **< 0.0001** |
| **Genistein, mean (SE), mg/d** | **1.06(0.12)** | **0.00(0.00)** | **0.01(0.00)** | **3.06(0.35)** | **< 0.0001** |
| **Glycitein, mean (SE), mg/d** | **0.15(0.02)** | **0.00(0.00)** | **-** | **0.54(0.06)** | **< 0.0001** |
| **Cyanidin, mean (SE), mg/d** | **2.52(0.16)** | **0.03(0.00)** | **0.59(0.01)** | **6.09(0.40)** | **< 0.0001** |
| **Petunidin, mean (SE), mg/d** | **1.11(0.13)** | **0.00(0.00)** | **0.04(0.00)** | **2.97(0.29)** | **< 0.0001** |
| **Delphinidin, mean (SE), mg/d** | **1.53(0.19)** | **0.00(0.00)** | **0.06(0.00)** | **3.95(0.39)** | **< 0.0001** |
| **Malvidin, mean (SE), mg/d** | **4.79(0.46)** | **0.00(0.00)** | **0.19(0.01)** | **12.79(0.74)** | **< 0.0001** |
| **Pelargonidin, mean (SE), mg/d** | **1.58(0.15)** | **0.00(0.00)** | **0.04(0.00)** | **4.27(0.33)** | **< 0.0001** |
| **Peonidin, mean (SE), mg/d** | **1.64(0.16)** | **0.00(0.00)** | **0.11(0.00)** | **4.15(0.42)** | **< 0.0001** |
| **Catechin, mean (SE), mg/d** | **7.79(0.26)** | **1.03(0.04)** | **4.53(0.04)** | **14.37(0.30)** | **< 0.0001** |
| **Epigallocatechin, mean (SE), mg/d** | **16.36(1.00)** | **0.03(0.00)** | **0.37(0.01)** | **35.01(1.48)** | **< 0.0001** |
| **Epicatechin, mean (SE), mg/d** | **10.09(0.29)** | **0.96(0.04)** | **5.88(0.09)** | **20.51(0.44)** | **< 0.0001** |
| **Epicatechin_3_gallate, mean (SE), mg/d** | **10.73(0.68)** | **0.00(0.00)** | **0.03(0.00)** | **25.17(1.06)** | **< 0.01** |
| **Epigallocatechin_3_gallate, mean (SE), mg/d** | **28.49(1.75)** | **0.00(0.00)** | **0.11(0.00)** | **65.54(2.66)** | **< 0.01** |
| **Theaflavin, mean (SE), mg/d** | **1.61(0.11)** | **0.00(0.00)** | **-** | **4.51(0.20)** | **< 0.0001** |
| **Thearubigins, mean (SE), mg/d** | **93.33(5.98)** | **0.00(0.00)** | **-** | **261.62(10.01)** | **< 0.0001** |
| **Eriodictyol, mean (SE), mg/d** | **0.18(0.01)** | **0.00(0.00)** | **0.02(0.00)** | **0.48(0.02)** | **< 0.0001** |
| **Hesperetin, mean (SE), mg/d** | **9.14(0.43)** | **0.00(0.00)** | **1.08(0.04)** | **28.99(0.82)** | **< 0.0001** |
| **Naringenin, mean (SE), mg/d** | **3.24(0.23)** | **0.01(0.00)** | **0.40(0.01)** | **9.45(0.57)** | **< 0.0001** |
| **Apigenin, mean (SE), mg/d** | **0.25(0.04)** | **0.01(0.00)** | **0.05(0.00)** | **0.44(0.07)** | **< 0.0001** |
| **Luteolin, mean (SE), mg/d** | **0.71(0.03)** | **0.05(0.00)** | **0.28(0.00)** | **1.35(0.05)** | **< 0.0001** |
| **Isorhamnetin, mean (SE), mg/d** | **0.90(0.04)** | **0.03(0.00)** | **0.33(0.00)** | **1.65(0.06)** | **< 0.0001** |
| **Kaempferol, mean (SE), mg/d** | **4.51(0.16)** | **0.36(0.01)** | **1.62(0.02)** | **7.90(0.16)** | **< 0.0001** |
| **Myricetin, mean (SE), mg/d** | **1.48(0.06)** | **0.10(0.00)** | **0.44(0.01)** | **2.73(0.08)** | **< 0.0001** |
| **Quercetin, mean (SE), mg/d** | **11.94(0.35)** | **2.53(0.04)** | **6.69(0.05)** | **18.91(0.49)** | **< 0.0001** |
| **Theaflavin_3_3_digallate, mean (SE), mg/d** | **1.78(0.12)** | **0.00(0.00)** | **-** | **4.98(0.22)** | **< 0.0001** |
| **Theaflavin_3q_gallate, mean (SE), mg/d** | **1.50(0.10)** | **0.00(0.00)** | **-** | **4.21(0.19)** | **< 0.0001** |
| **Theaflavin_3_gallate, mean (SE), mg/d** | **1.28(0.09)** | **-** | **-** | **-** | **-** |
| **Gallocatechin, mean (SE), mg/d** | **1.72(0.11)** | **0.00(0.00)** | **0.02(0.00)** | **3.35(0.15)** | **< 0.0001** |
| **Subtotal_Catechins, mean (SE), mg/d** | **75.19(3.95)** | **2.83(0.09)** | **13.48(0.19)** | **160.06(5.94)** | **< 0.0001** |

Note: The flavonoids were divided into Q1, Q2 and Q3 by tertile. SE: standard error. Data were analyzed using ANOVA analysis. 

**Table S2. The averages of biological age (BA) and its differences with chronological age (CA) by quintiles of total flavonoid intake.**

| **Variables** | **BA** | | **BA Difference (BA-CA)** | | **Heart BA** | | **Heart BA Difference (BA-CA)** | | **Kidney BA** | | **Kidney BA Difference (BA-CA)** | | **Liver**  **BA** | | **Liver BA Difference (BA-CA)** | |
| --- | --- | --- | --- | --- | --- | --- | --- | --- | --- | --- | --- | --- | --- | --- | --- | --- |
|  | **Mean (SD)** | **P** | **Mean (SD)** | **P** | **Mean (SD)** | **P** | **Mean (SD)** | **P** | **Mean (SD)** | **P** | **Mean (SD)** | **P** | **Mean (SD)** | **P** | **Mean (SD)** | **P** |
| **Isoflavones** |  |  |  |  |  |  |  |  |  |  |  |  |  |  |  |  |
| **Q1** | **48.58(0.55)** | **<0.01** | **0.07(0.22)** | **<0.01** | **48.33(0.55)** | **<0.01** | **-0.19(0.25)** | **<0.01** | **49.31(0.78)** | **<0.01** | **0.80(0.58)** | **0.55** | **47.76(1.26)** | **<0.01** | **-0.75(1.14)** | **<0.01** |
| **Q2** | **49.67(0.77)** |  | **-0.81(0.19)** |  | **49.36(0.90)** |  | **-1.12(0.37)** |  | **50.58(0.81)** |  | **0.10(0.50)** |  | **45.84(1.54)** |  | **-4.64(1.38)** |  |
| **Q3** | **43.59(0.62)** |  | **-1.13(0.21)** |  | **43.04(0.57)** |  | **-1.68(0.28)** |  | **44.89(0.76)** |  | **0.17(0.40)** |  | **39.93(1.11)** |  | **-4.78(0.77)** |  |
| **Anthocyanidins** |  |  |  |  |  |  |  |  |  |  |  |  |  |  |  |  |
| **Q1** | **45.13(0.57)** | **<0.01** | **0.54(0.23)** | **<0.01** | **44.56(0.61)** | **<0.01** | **-0.03(0.36)** | **<0.01** | **46.83(0.71)** | **0.15** | **2.24(0.52)** | **<0.01** | **45.20(1.21)** | **0.69** | **0.61(1.31)** | **<0.01** |
| **Q2** | **47.09(0.73)** |  | **-0.60(0.20)** |  | **46.97(0.74)** |  | **-0.72(0.28)** |  | **48.19(0.84)** |  | **0.49(0.47)** |  | **44.73(1.28)** |  | **-2.97(1.02)** |  |
| **Q3** | **48.47(0.75)** |  | **-1.45(0.20)** |  | **48.05(0.72)** |  | **-1.87(0.37)** |  | **48.80(0.86)** |  | **-1.11(0.58)** |  | **43.60(1.45)** |  | **-6.31(1.07)** |  |
| **Flavan_3_ols** |  |  |  |  |  |  |  |  |  |  |  |  |  |  |  |  |
| **Q1** | **44.67(0.74)** | **0.01** | **0.07(0.23)** | **<0.01** | **44.25(0.71)** | **<0.01** | **-0.35(0.27)** | **<0.01** | **45.54(0.91)** | **0.03** | **0.95(0.52)** | **0.39** | **43.82(1.45)** | **0.39** | **-0.78(1.11)** | **0.02** |
| **Q2** | **47.91(0.81)** |  | **-0.55(0.26)** |  | **48.03(0.82)** |  | **-0.42(0.45)** |  | **48.46(1.01)** |  | **0.01(0.58)** |  | **43.57(1.67)** |  | **-4.89(1.22)** |  |
| **Q3** | **48.12(0.68)** |  | **-1.07(0.17)** |  | **47.45(0.69)** |  | **-1.75(0.28)** |  | **49.46(0.86)** |  | **0.26(0.49)** |  | **45.42(1.01)** |  | **-3.77(0.81)** |  |
| **Flavanones** |  |  |  |  |  |  |  |  |  |  |  |  |  |  |  |  |
| **Q1** | **47.02(0.73)** | **<0.01** | **0.01(0.27)** | **<0.01** | **46.21(0.72)** | **<0.01** | **-0.80(0.34)** | **0.54** | **48.65(0.93)** | **0.08** | **1.64(0.64)** | **<0.01** | **47.08(1.36)** | **0.06** | **0.07(0.98)** | **<0.01** |
| **Q2** | **45.51(0.62)** |  | **-0.61(0.20)** |  | **44.99(0.63)** |  | **-1.13(0.29)** |  | **46.80(0.84)** |  | **0.67(0.49)** |  | **42.98(1.21)** |  | **-3.14(1.02)** |  |
| **Q3** | **49.09(0.81)** |  | **-1.06(0.17)** |  | **49.27(0.89)** |  | **-0.87(0.37)** |  | **49.11(0.74)** |  | **-1.04(0.44)** |  | **44.11(1.33)** |  | **-6.03(1.14)** |  |
| **Flavones** |  |  |  |  |  |  |  |  |  |  |  |  |  |  |  |  |
| **Q1** | **45.52(0.90)** | **0.12** | **0.59(0.22)** | **<0.01** | **45.03(1.03)** | **0.04** | **0.09(0.42)** | **0.03** | **45.53(1.04)** | **0.03** | **0.60(0.60)** | **0.41** | **47.89(1.69)** | **0.01** | **2.95(1.11)** | **<0.01** |
| **Q2** | **46.82(0.65)** |  | **-0.40(0.22)** |  | **46.16(0.64)** |  | **-1.06(0.34)** |  | **48.11(0.91)** |  | **0.89(0.67)** |  | **45.67(1.25)** |  | **-1.55(1.08)** |  |
| **Q3** | **47.81(0.67)** |  | **-1.20(0.18)** |  | **47.67(0.68)** |  | **-1.34(0.29)** |  | **48.98(0.77)** |  | **-0.03(0.35)** |  | **42.13(1.09)** |  | **-6.89(0.87)** |  |
| **Flavonols** |  |  |  |  |  |  |  |  |  |  |  |  |  |  |  |  |
| **Q1** | **47.89(1.69)** | **0.01** | **0.00(0.24)** | **0.01** | **45.99(0.84)** | **0.74** | **-0.54(0.39)** | **0.11** | **46.75(1.05)** | **0.19** | **0.22(0.79)** | **0.26** | **49.17(1.59)** | **<0.01** | **2.64(1.32)** | **<0.01** |
| **Q2** | **45.67(1.25)** |  | **-0.46(0.25)** |  | **46.70(0.90)** |  | **-0.63(0.43)** |  | **47.06(0.88)** |  | **-0.26(0.63)** |  | **44.52(1.23)** |  | **-2.80(0.94)** |  |
| **Q3** | **42.13(1.09)** |  | **-0.85(0.18)** |  | **46.85(0.74)** |  | **-1.30(0.27)** |  | **49.00(0.90)** |  | **0.85(0.41)** |  | **42.79(1.12)** |  | **-5.36(0.81)** |  |

**Note:** Values are means (SD) for continuous variables; SD: standard deviation. The flavonoids were divided into Q1, Q2 and Q3 by tertile. Data were analyzed using χ^2^ tests for the the categorical variables, **and** data were analyzed using ANOVA analysis for continuous variables.

**Table S3 The results of multiple linear regression analysis for the association between flavonoid intake and differences in biological age (BA) with chronological age (CA).**

| **Variables** | **Overall BA Differences** | | **Heart BA Differences** | | **Kidney BA Differences** | | **Liver BA Differences** | |
| --- | --- | --- | --- | --- | --- | --- | --- | --- |
|  | **β(95%CI)** | **P** | **β(95%CI)** | **P** | **β(95%CI)** | **P** | **β(95%CI)** | **P** |
| **Isoflavones** |  |  |  |  |  |  |  |  |
| **Q1** | **Ref** | **-** | **Ref** | **-** | **Ref** | **-** | **Ref** | **-** |
| **Q2** | **-0.88(-1.41, -0.35)** | **<0.01** | **-0.93(-1.63, -0.22)** | **0.01** | **-0.7(-2.15, 0.75)** | **0.33** | **-3.88(-7.28, -0.49)** | **0.03** |
| **Q3** | **-1.19(-1.71, -0.68)** | **<0.01** | **-1.49(-2.14, -0.84)** | **<0.01** | **-0.63(-1.89, 0.64)** | **0.32** | **-4.03(-6.40, -1.66)** | **<0.01** |
| **Anthocyanidins** |  |  |  |  |  |  |  |  |
| **Q1** | **Ref** | **-** | **Ref** | **-** | **Ref** | **-** | **Ref** | **-** |
| **Q2** | **-1.14(-1.73, -0.55)** | **<0.01** | **-0.69(-1.55, 0.16)** | **0.11** | **-1.75(-3.07, -0.43)** | **0.01** | **-3.57( -6.53, -0.62)** | **0.02** |
| **Q3** | **-1.99(-2.48, -1.49)** | **<0.01** | **-1.84(-2.83, -0.86)** | **<0.01** | **-3.36(-4.55, -2.16)** | **<0.01** | **-6.92(-10.35, -3.49)** | **<0.01** |
| **Flavan_3_ols** |  |  |  |  |  |  |  |  |
| **Q1** | **Ref** | **-** | **Ref** | **-** | **Ref** | **-** | **Ref** | **-** |
| **Q2** | **-0.62(-1.21, -0.03)** | **0.04** | **-0.07(-1.13, 0.98)** | **0.89** | **-0.94(-2.37, 0.49)** | **0.19** | **-4.11(-7.02, -1.20)** | **0.01** |
| **Q3** | **-0.62(-1.21, -0.03)** | **<0.01** | **-1.4(-2.17, -0.63)** | **<0.01** | **-0.69(-2.09, 0.72)** | **0.33** | **-3(-5.63, -0.36)** | **0.03** |
| **Flavanones** |  |  |  |  |  |  |  |  |
| **Q1** | **Ref** | **-** | **Ref** | **-** | **Ref** | **-** | **Ref** | **-** |
| **Q2** | **-0.63(-1.23, -0.03)** | **0.04** | **-0.33(-1.12, 0.45)** | **0.39** | **-0.97(-2.42, 0.49)** | **0.18** | **-3.22(-5.92, -0.51)** | **0.02** |
| **Q3** | **-1.07(-1.65, -0.49)** | **<0.01** | **-0.07(-1.14, 1.00)** | **0.89** | **-2.67(-4.13, -1.22)** | **<0.01** | **-6.11(-9.34, -2.87)** | **<0.01** |
| **Flavones** |  |  |  |  |  |  |  |  |
| **Q1** | **Ref** | **-** | **Ref** | **-** | **Ref** | **-** | **Ref** | **-** |
| **Q2** | **-0.99(-1.50, -0.47)** | **<0.01** | **-1.15(-2.16, -0.13)** | **0.03** | **0.3(-1.19, 1.78)** | **0.69** | **-4.5( -7.23, -1.76)** | **<0.01** |
| **Q3** | **-1.79(-2.33, -1.24)** | **<0.01** | **-1.43(-2.51, -0.35)** | **0.01** | **-0.63(-1.78, 0.52)** | **0.27** | **-9.84(-12.53, -7.15)** | **<0.01** |
| **Flavonols** |  |  |  |  |  |  |  |  |
| **Q1** | **Ref** | **-** | **Ref** | **-** | **Ref** | **-** | **Ref** | **-** |
| **Q2** | **-0.46(-1.11, 0.18)** | **0.15** | **-0.09(-1.28, 1.10)** | **0.88** | **-0.48(-2.62,1.65)** | **0.65** | **-5.44( -7.94, -2.94)** | **<0.01** |
| **Q3** | **-0.86(-1.36, -0.36)** | **<0.01** | **-0.77(-1.61, 0.08)** | **0.07** | **0.63(-1.08,2.33)** | **0.46** | **-8.01(-10.81, -5.20)** | **<0.01** |

Note: The flavonoids were divided into Q1, Q2 and Q3 by tertile. CI, confidence interval. Linear regression analysis was used to determine the standard β coefficient. Data were analyzed using ANOVA analysis for continuous variables.
